# Pest-removal services provided by birds on subsistence farms in south-eastern Nigeria

**DOI:** 10.1371/journal.pone.0255638

**Published:** 2021-08-09

**Authors:** Murna Tela, Will Cresswell, Hazel Chapman

**Affiliations:** 1 Department of Biological Sciences, Gombe State University, Gombe, Nigeria; 2 School of Biological Sciences, University of Canterbury, Christchurch, New Zealand; 3 School of Biology, University of St Andrews, St Andrews, United Kingdom; National University Comahue, ARGENTINA

## Abstract

To what extent birds provide the ecosystem service of pest control in subsistence farms, and how this service might depend on retained natural habitats near farmlands is unexplored in West Africa. To fill this knowledge gap, we placed plasticine mimics of insect pests on experimentally grown crops on the Mambilla Plateau, South Eastern Nigeria. We recorded bird attacks on the mimics and the proportion of mimics removed by birds. We also determined the influence of distance of crops from forest fragments on both attack and removal rates. We placed 90 potted plants of groundnut (*Arachis hypogea*) and bambara nut (*Vigna subterranea*) along 15 transects running 4.5 km from forest edge into open grassland. Each plant had six of the 540 mimics in total placed on their leaves. We inspected the potted plants weekly for 12 weeks to record (i) the presence of bird beak marks on mimics, and (ii) the number of missing mimics. Once a week we collected all the mimics from the plants and counted the number of assumed beak marks. After counting we replaced the mimics on the plants, mark free. We found a strong positive correlation between the abundance of insectivorous birds and the mean number of missing mimics and/or bird attack marks on mimics. However, this positive effect of insectivorous bird abundance on prey mimic attack/removal became less strong the farther they were from a forest fragment. We found increased predation rates and abundance of insectivorous birds closer to forest fragments. Our data suggest that pest predation may be a key ecosystem service provided by insectivorous birds on Nigerian farmlands. Farmlands that are closer to forest fragments may experience a higher rate of pest control by insectivorous birds than those further away, suggesting that retaining forest fragments in the landscape may enhance pest control services in sub-Saharan subsistence farms.

## Introduction

Across Africa, subsistence farming, whereby farmers produce food sufficient only for their own use, is the most widespread type of agriculture. This is especially so in West Africa, where most farms range between 1–5 ha [1] and are located in rural areas, typically scattered within savannah or forest habitats. While historically there was some sort of equilibrium between farms and more natural habitat, this is being lost. Africa’s rapidly increasing human population [2], and associated food requirements is resulting in a decline in forest cover as trees are cleared to create more farmland. Forests are increasingly fragmented and woody corridors linking forest fragments are being destroyed [3, 4]. This land use change has a plethora of negative effects on the natural environment, including reduced biodiversity and genetic diversity [5]. Fenta et al. [6] quantified this loss of ecosystem service provision in natural African landscapes and warned of the negative long-term effects of such loss on climate regulation and nursery services.

The value of birds in controlling insect pests, and of forest fragments in maintaining bird populations is acknowledged in tropical agroecosystems [7–9]. However, to what extent birds positively control insect pest in African subsistence farms remains mostly unknown. Many farmers perceive birds as pests [10, 11]. Thus, it is extremely important that the net effect of birds as insect predators in farmlands is quantified. If the economic impact of bird predation in controlling insect pests is positive, farmers will benefit by ensuring they manage their farmland in ways which provide for bird habitat [12]. A vital first step in the process must be quantifying the ecosystem services birds provide.

Across Africa, insect pests threaten both food security and farmers’ income [13–16], accounting for almost 58% of food insecurity in West Africa [16, 17]. Subsistence farmers are especially vulnerable and suffer significant loss of yield from pre-harvest insect pests [18–21]. The Lepidopteran stem borer (Noctuidae: *Busseola fusca*) and the fall armyworm (Noctuidae: *Spodoptera frugiperda*) are key pests, accounting for up to 75% of crop yield losses [22–26]. More specifically, in Nigeria about 30% of cocoa yield loss is caused by the brown cocoa mirid (Miridae: *Sahlbergella singularis*) [27].

Despite their negative effects, African subsistence farmers rarely control crop pests because of lack of availability and/or cost of pesticides [16, 28]. Moreover, the use of chemicals is problematic; insecticides are commonly applied without regard to safety precautions [13], with major implications for human health, the ecosystem and the environment [29]. The regular use of pesticides can lead to pest resistance [30], harm insect pollinators and destroy food resources of insect predators such as birds [31–33].

An alternative to the use of pesticides is natural pest control and insectivorous birds, for example, provide an economically and environmentally sound alternative in many systems [31, 32, 34, 35]. In both temperate and tropical locations [9, 36–38] predation of pests by birds can substantially benefit crop plants [7, 39, 40]. Zhang et al. [16] estimate this service to add between 100–400 billion USD per annum to the world’s economy. In Costa Rica, Karp et al. [8] showed birds were directly responsible for an increase in coffee yield worth US$310 ha^-1^ through predating the coffee berry borer (*Hypothenemus hampei*). Tropical studies have repeatedly linked the success of birds as predators of insect pests in agricultural landscapes with native forest proximity [8, 33, 41–43]; birds use forest fragments as sites for breeding, hibernation and roosting [44]. Thus, maintaining forest fragments within an agricultural landscape may help improve insect pest control services by birds. For example, in Central Sulawesi, the lemon-bellied white-eye (*Zosterops chloris*) occurs in both coffee plantation and forest habitats [9]. It plays an important role in suppressing insect pests in the coffee plantation, and control is enhanced by the plantation’s proximity to forest [41].

The agricultural landscape in south-eastern Nigeria consists of a mosaic of small subsistence farms with annual crops such as maize (*Zea mays*), groundnuts (*Arachis hypogea*) and bambara nuts (*Vigna subterranea*), with patches of forest at varying distances from the crop fields [45]. Groundnuts are a major source of food and cooking oil in Nigeria [46, 47] and across West Africa, and often generate income for women [48]. In Nigeria, insect pests can reduce groundnut yield by up to 40% [49], although a loss of between 10–20% is the norm [48]. Foliar pests leading to these reductions in crop yield include the (Scarabaeidae: *Lachnosterna quercina*), false wireworms (Tenebrionidae: *Gonocephalum* spp.) and groundnut hopper (Cicadellidae: *Hilda patruelis*; [47, 48, 50, 51]). But given evidence from other tropical systems, these subsistence landscapes may harbour insectivorous bird species in forest patches that may reduce invertebrate pests of crops and thus benefit farmers. Moreover, as many forest birds may not fly far from forest [52], the efficacy of control may be influenced by the proximity of farm to forest. If this is the case it is essential for conservation that this is known to farmers.

Quantifying pest control by birds is difficult because birds forage quickly and most predation leaves no trace [53]. One method is to attach plasticine prey mimics (e.g., caterpillars) to crop plants and record their removal rate from the plants and/or the number of attack marks left behind in the plasticine [54–57]. Different coloured plasticine can be shaped to resemble specific prey species [58, 59], and predators can be determined, at least to a higher taxanomic level, by the marks they leave behind [58–60].

Here, we use artificial plasticine models in West African subsistence farmlands to test if: i) crop pests are attacked by insect-eating bird abundance and ii) this predation rate depends on the proximity of farmland to forest fragments. We made two main predictions:

1. Predation rate (bird attack marks or missing pest mimics) will i) correlate positively with insectivore abundance, and negatively with distance to forest fragments, and ii) the strength of the positive effect of bird abundance on predation rates will decrease with increasing distance from forest fragments.
2. Insect-eating bird abundance will correlate negatively with increasing distance to a forest fragment.

## Materials and methods

### Study area

**Fig 1:**
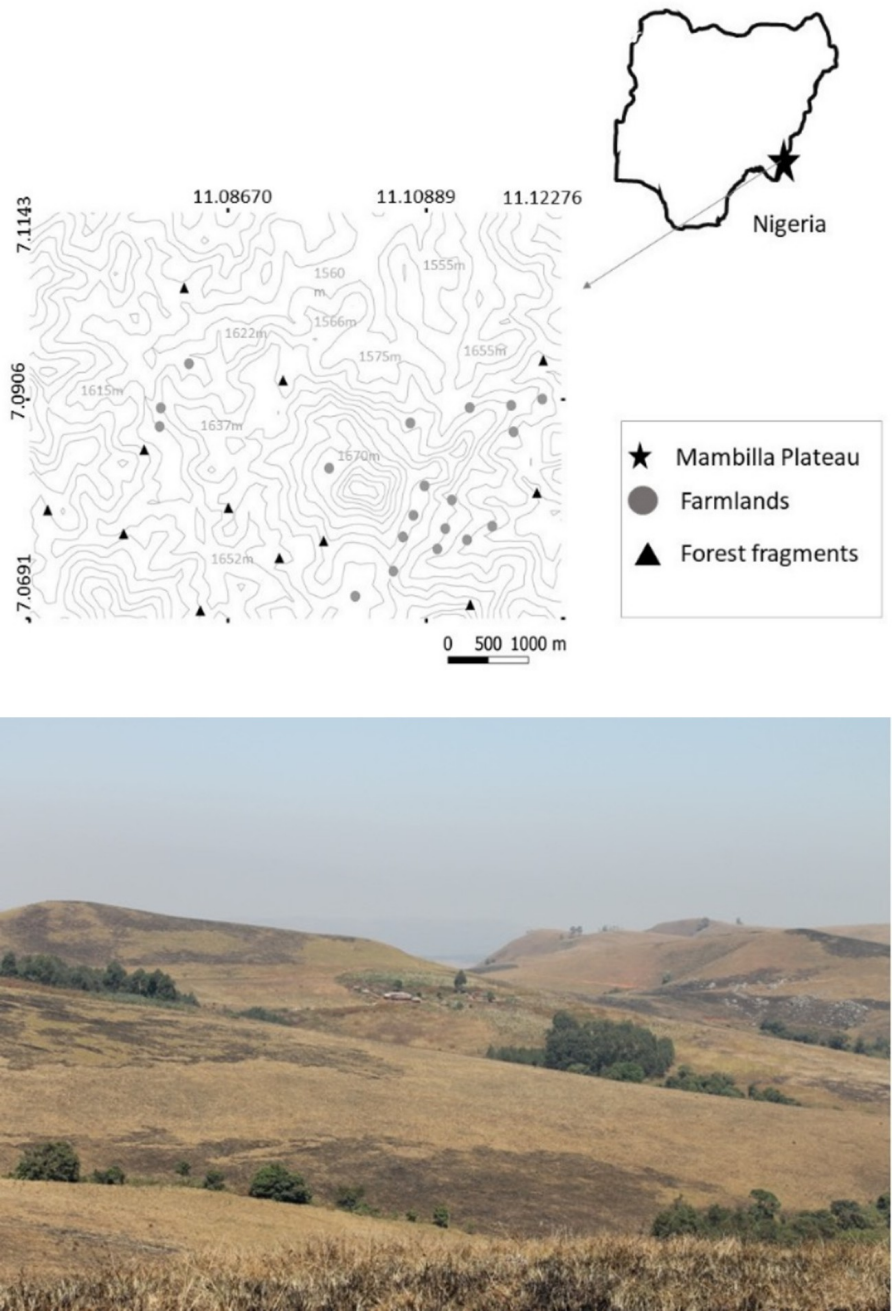
The study area. (A) Map of Nigeria and the location of the experimental site on Mambilla Plateau, Nigeria. (B) The study landscapes.

Our study area is located within and beyond the Ngel Nyaki forest reserve [61], along the north-western escarpment of the Mambilla plateau in Taraba State (7.16°N, 11.66°E), south-eastern Nigeria (Fig 1A). It includes Yelwa village and is close to Nigerian Montane Forest Project (NMFP) field station. The plateau, with an average elevation of 1,600 m is characterised by gently undulating hills mostly covered in overgrazed *Sporobilis* grassland [61]. Small forest fragments, mostly degraded and grazed by cattle, line the side of streams. Ngel Nyaki forest, about 7.2 km^2^ in area is the largest stand of submontane forest on the plateau. It is confined to the steep slopes of an ancient volcano, with the tops of trees adjacent to the grassland (Fig 1B) and is rich in bird and animal life [61]. Small subsistence farms surround Yelwa village and spread out along the bottom of stream-side valleys. Annual crops include maize, groundnuts, bambara nuts, ginger (*Aframomum melegueta*), kidney beans (*Phaseolus vulgaris*), potatoes (*Solanum tuberosum*) and yam (*Dioscorea rotundata*). The Mambilla plateau has a distinct wet (April-September) and dry season (October-March) with a mean annual rainfall from 1600–2000 mm [62]. The minimum average monthly temperature ranges from 15.5–18.5 ⁰C and the maximum from 27.5–30.5 ⁰C [61].

### Experiments

We used groundnuts and bambara nuts as our focal crop species because they are common on the Mambilla plateau and are widespread across the savanna regions of Africa [45, 50, 63].

**Fig 2:**
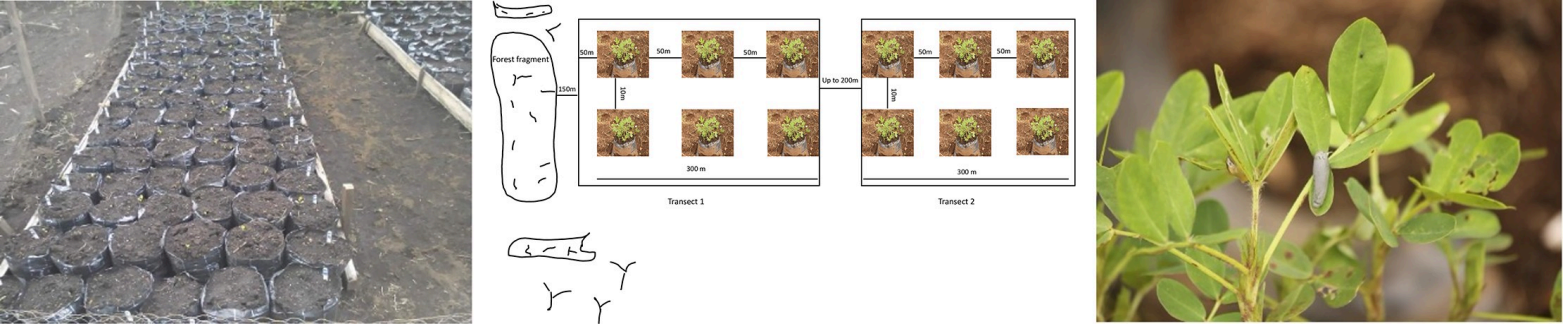
Experimental set-up. (A) seeds of groundnuts and bambara nuts planted into polypots. (B) Experimental set up in farmlands; six crop plants were placed in each transect (i.e., three replications for each crop species). (C) Mimics placed on crop plant.

We bought seeds of bambara nuts and groundnuts from Nguroji village local market and planted into garden-soil-filled polypots measuring 32 × 40 cm in the nursery of the Nigerian Montane Forest Project (Fig 2A). Each seed species was planted into 45 polypots making a total of 90 polypots (three seeds/bag). The seeds were protected from birds and rodents using a cage of wire netting with pore size of 0.8 mm x 1.2 mm. The seeds germinated approximately 10 days after sowing, with germination of all seeds complete after 15 days. Three weeks after planting, we moved them into 10 farmlands and placed them along 15 line transects, with each transect measuring 300 m, making a total of 4500 m of transects. Five farmlands had two transects each and the other five farmlands had one transect each, depending on the size of farmland. The distance between transects was at least 150 m. We placed one groundnut plant and one bambara nut plant in each section, with a minimum increasing distance of 50 m across sections. In total, six crop plants (i.e., three replications for each crop species) were placed in each transect (Fig 2B).

To estimate bird predation rates, we used plasticine, a non-toxic putty-like modelling material (Newplast, UK). We made plasticine mimics of brown and green caterpillars, grey beetles and grey larvae as baits for birds [37, 60, 64, 65]. The colours and shape of the plasticine mimics resembled as closely as possible insect species we had found on the crop plants in the fields. We tried three different prey types to maximise the chance that at least one of the mimic types was considered as prey. At the end of the experiment there was found to be no difference in predation rates across mimic type: inclusion of prey type in the models had no significant effect (footnotes to the models in Tables 1 and 2). Caterpillars, beetles and larvae were all made to the same size; diameter 3.5 mm and length 25 mm and as close to the shape of their real-life counterparts as possible. We then attached the mimics to the plant leaves using UHU all-purpose adhesive glue (Fig 2C). We randomly placed six mimics on the leaves of each experimental crop plant, one mimic per leaf, amounting to a total of 540 mimics. We note that placing mimics on every plant may not be ideal. For example, birds have a good learning ability so may learn that the mimics are not prey [37, 54, 66]. We monitored the mimics daily for 12 weeks along transect sections (three transects per day) to record bird attack marks and/or the number of missing mimics. At the end of each week, all mimics were collected for identification of bird predation marks, then remoulded into prey shapes and replaced on the crop plants. Bird attack marks were identified under a magnifying hand lens. Artificial mimics were considered predated if they had a distinct beak mark that looked like a bird attack mark, or they were missing. If an entire pest mimics was removed from the plant, we considered it bird predation, because most vertebrate attacks on artificial caterpillars are by birds [54], and predatory arthropods (e.g., wasps and other insects) may be unable to remove an entire mimics from plant, only pieces of the mimics, due to the glued portion of mimics [67]. Therefore, we considered all missing caterpillars to have been predated by birds, although we cannot exclude the possibility that some were removed by other taxa such as mammals.

**Table 1:** Model 1A. The relationship between bird attack marks on pest mimics and insect-eating bird abundance, and distance to a forest fragment. The model also tests how the relationship between bird attack marks and bird abundance depends on distance to a forest fragment.

Variables	Estimate	SE	*t*	*p*
(Intercept)	3.47	0.29	12.14	**<0.001**
Distance	−0.00044	0.00010	−4.57	**<0.001**
Insect-eating bird abundance	0.081	0.013	6.12	**<0.001**
Distance*Insect-eating birds	−0.000019	0.000010	−1.87	0.063

*Note*. Full model; Bird attack marks = insect-eating birds + distance + insect-eating birds*distance, random = ~1|section, method = "ML". Significant p–values are given in bold. Pest mimic type was not significant when added to this model: Green mimics -0.064 ± 0.17, t = -0.4, P = 0.70; grey mimics -0.066 ± 0.17, t = -0.4, P = 0.70, brown mimics set to the intercept.

**Table 2:** Model 1B. The relationship between number of missing pest mimics and insect-eating bird abundance, distance to a forest fragment. The model also tests how the relationship between missing pest mimics and bird abundance depends on distance to a forest fragment.

Variables	Estimate	SE	*t*	*p*
(Intercept)	0.85	0.43	1.98	**0.052**
Distance	0.00022	0.00019	1.16	0.248
Insect-eating bird abundance	0.085	0.023	3.67	**0.005**
Distance*Insect-eating birds	−0.000028	0.000011	−2.52	**0.014**

*Note*. Full model; Missing pest mimics = insect-eating birds + distance + insect-eating birds*distance, random = ~1|section, method = "ML". Significant p–values are given in bold. Pest mimic type was not significant when added to this model: Green mimics 0.024 ± 0.26, t = 0.09, P = 0.92; grey mimics -0.08 ± 0.25, t = -0.3, P = 0.76, brown mimics set to the intercept.

To determine insectivorous bird abundance, we conducted 10-minute point counts of birds in each section of the 4500-m transect. In each section, survey points were located a minimum of 50 m apart and spatially overlapped with the plasticine mimics’ placement area following the methodology of Roels et al. [58]. Each section was surveyed four times per week by the same observers between 6:30 am and 10:30 am. During each survey, all birds detected by sight or sound within a 25-m radius were recorded. Birds flying through or over the count circle were not counted. Following the methodology of Wattel [68], all bird species that included insects as part of their diet, even though insects may not be a primary component of their diet, were included in the dataset. Thus, all species used for analysis were species that are capable of eating insects the size of our artificial mimics.

### Statistical analysis

We used linear mixed-effects models (LMMs) in R statistical software [69] to test i) whether the rate at which the mimics were attacked or removed by birds depended on abundance of birds, and ii) whether proximity to forest fragment affected the predation rate (i.e., number of bird attack marks or missing pest models).

The response variable was calculated per plant as the sum of all marks, or removed mimics, across all six mimics per plant. This gave an approximately normally distributed response variable, with most plants having some evidence of predation, and few with zero, so a Gaussian error distribution was assumed: the residuals from the final model were reasonably normally distributed.

We modelled the bird attack marks on pest mimics as a function of abundance of insect-eating birds (Model 1A); section (i.e. section of each transect) was included as a random effect because of how the experiment was designed, with distance from forest fragments and insect-eating bird abundance as a fixed effect, and included the interaction between distance from forest fragment and insect-eating bird abundance, to test whether the relationship between predation rate and distance was dependent on the presence of birds.

Our rationale was that if insect-eating birds are an important component of crop–pest predators, then we would expect higher predation rates to be associated with higher abundances of insect-eating birds. Moreover, if proximity to forest fragments increases the predation rate effect of insect-eating birds, then we would expect this effect to decrease with increasing distance from forest fragments (that is, a negative interaction between distance and insect-eating bird abundance). We graphically checked adequacy of models using model diagnostics plots. There was no evidence of non-linear effects (that is quadratic or logarithmic treatment of predictors did not improve model fits).

We constructed a second model using Model 1A but without distance, to test for an overall main effect of insect-eating bird on predation rates (Model 2A).

We constructed a third model using Model 1A but without abundance of insect-eating birds to test for an overall effect of proximity of the forest on predation rates (Model 3A).

Models 1B, 2B & 3B: We repeated each of the three models (1A, 1B and 1C) with the alternative index of predation rate—missing mimics (count of mimics that were not found).

We used a GLMM to test if forest proximity affected the abundance of insect-eating birds (Model 4). We modelled insect-eating bird as a function of distance from forest fragment; sections were included as random effects, with distance as a fixed effect.

## Results

In total, we recorded 1297 attack marks on pest mimics over the 12 weeks of our study, of which 1258 (97%) were bird attack marks (some of the mimics had more than one bird attack marks) and 39 (3%) were unidentified. One hundred and fifty-seven mimics were missing (15% of the total mimics). Eighty-six (8% of the total mimics) were damaged in a way that was unlikely to be related to bird predation and these were not included in the analyses. A total number of 4511 insect-eating birds were recorded from 97 species across the 15 transects (S5 Table). The 10 most abundant species or species groups were the family Cisticolidae (715), common bulbul *Pyconotus barbatus* (378), African stonechat *Saxicola torquatus* (291), bronze mannikin *Spermestes cucullate* (289), common fiscal *Lanius collaris* (282), little bee-eater *Merops pusillus* (243), whinchats *Saxicola rubetra* (190), rufous-naped lark *Mirafra africana* (137), northern grey-headed sparrow *Passer griseus* (123) and yellow-throated longclaw *Macronyx croceus* (121). Together, these 10 species represent 56% of the total individuals recorded.

**Fig 3:**
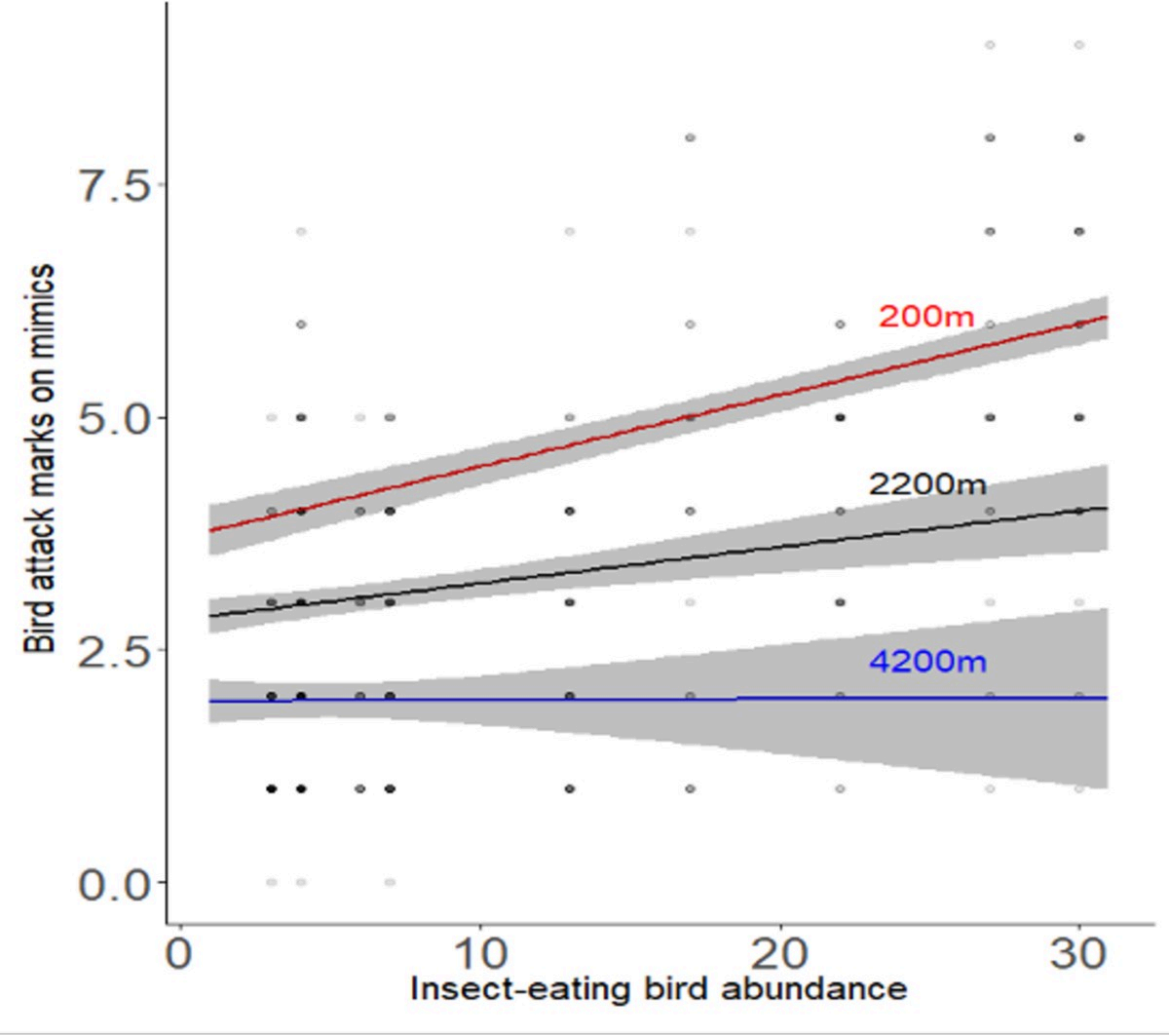
How the relationship between bird attack rates on prey mimics with insect-eating bird abundance varies with distance from a forest fragment. We estimated the predicted values from model in Table 1 and plotted the predicted lines. Crop was set to A and the colour to brown, and we plotted three separate lines for close distance at 200m, intermediate distance at 2200m and a far distance at 4200m. The gradient becomes less steep away from the forest fragments, showing that the positive effect of insect-eating bird abundance on prey mimics depends on proximity to forest.

**Fig 4:**
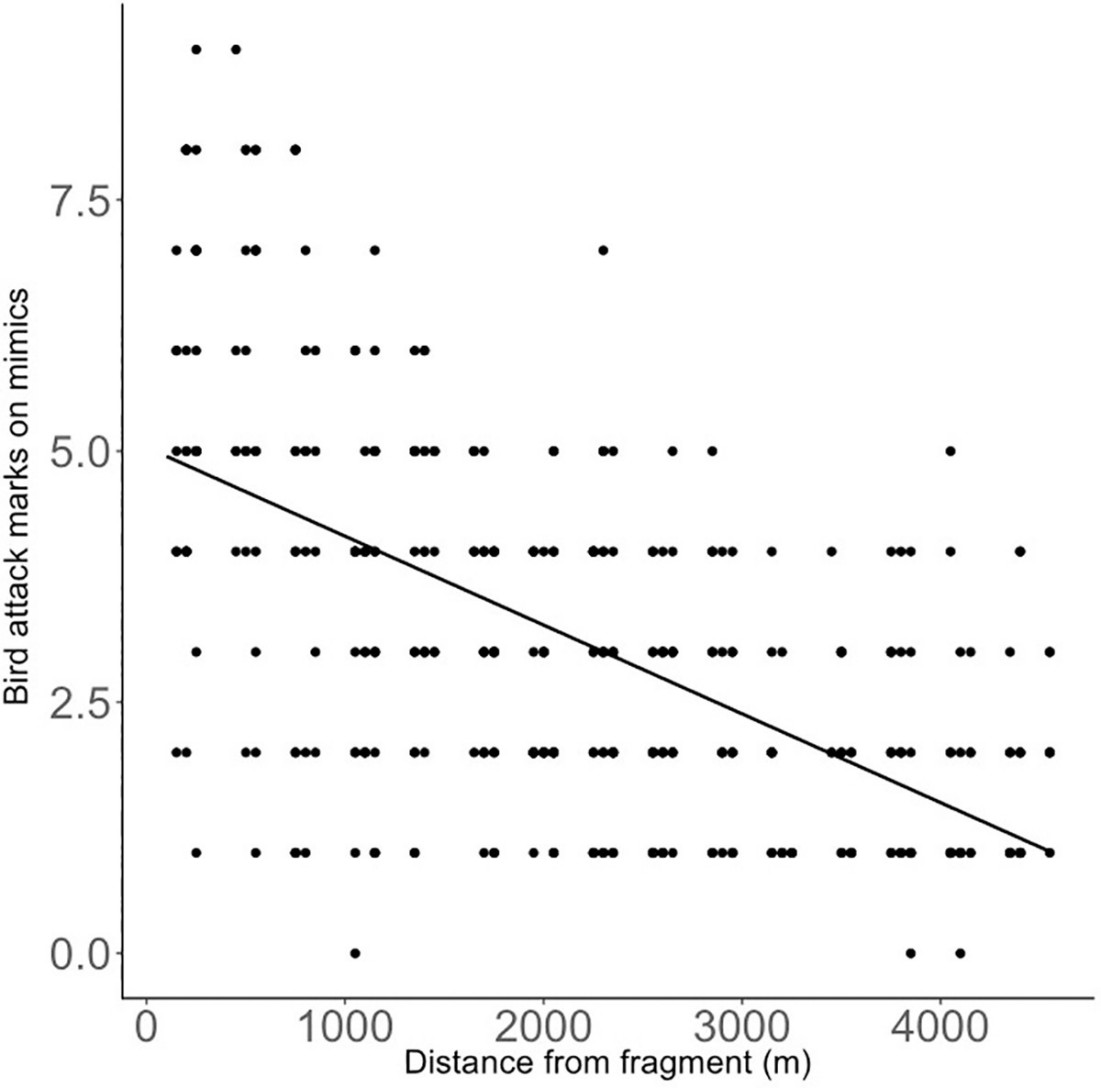
The relationship between bird attack marks on pest mimics and distance of crops from forest fragments. Predicted lines are plotted from the model in S2 Table.

The number of bird attack marks on the mimics increased with insect-eating bird abundance, but this positive relationship became weaker the further from the forest fragments (Table 1, Fig 3). We found a strong positive main effect of insect-eating bird abundance when not controlling for distance (S1 Table) and a strong negative effect of distance when not controlling for insect-eating bird abundance (S2 Table, Fig 4).

**Fig 5:**
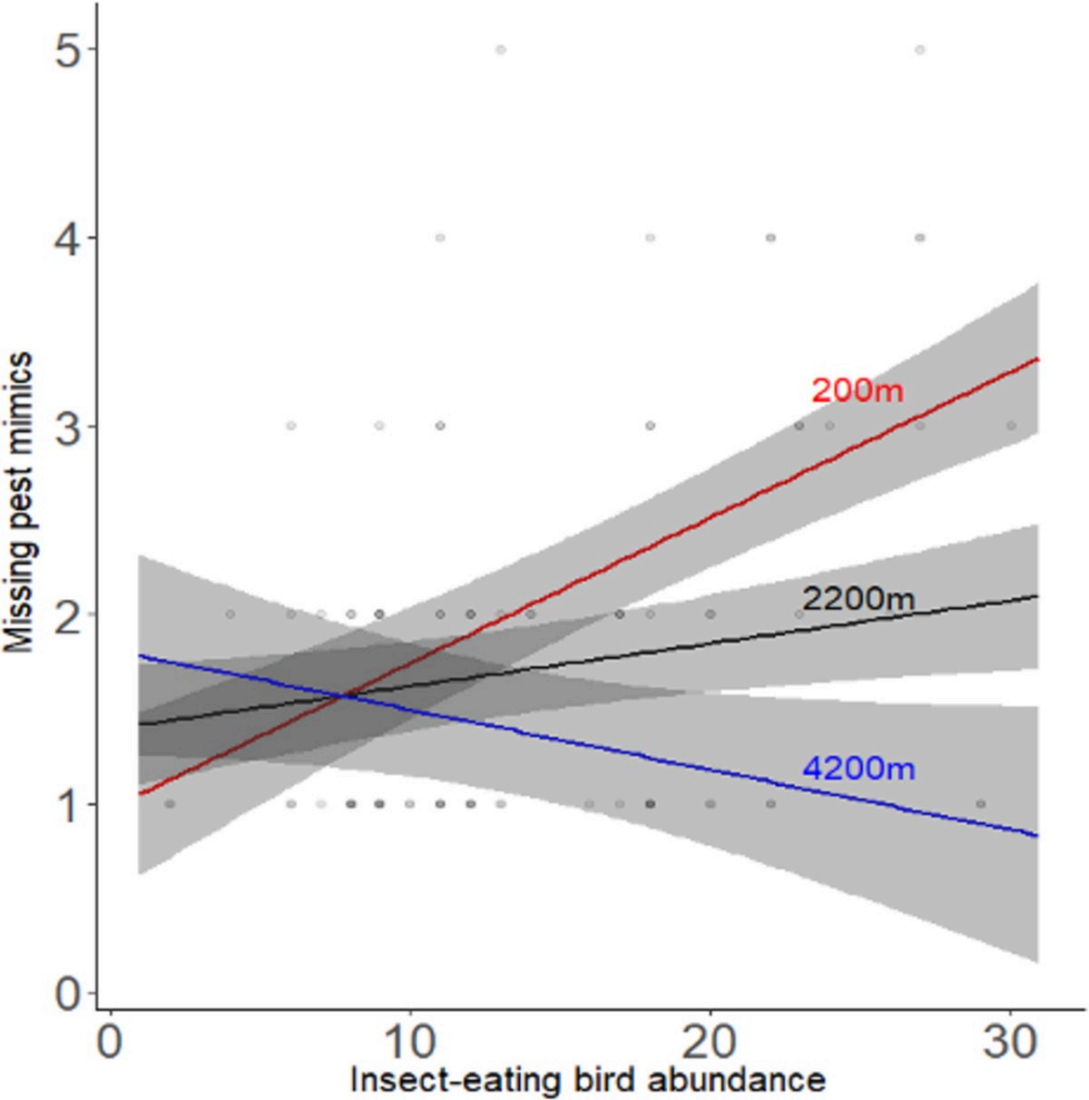
How the relationship between missing prey mimics and insect-eating bird abundance varies with distance from a forest fragment. We estimated the predicted values from model in Table 2 and plotted the predicted lines. Crop was set to A and the colour to brown, and we plotted three separate lines for close distance at ‘200m’, intermediate distance at ‘2200m’ and a far distance at ‘4200m’. These lines show how the gradient becomes less steep further away from the forest fragments.

**Fig 6:**
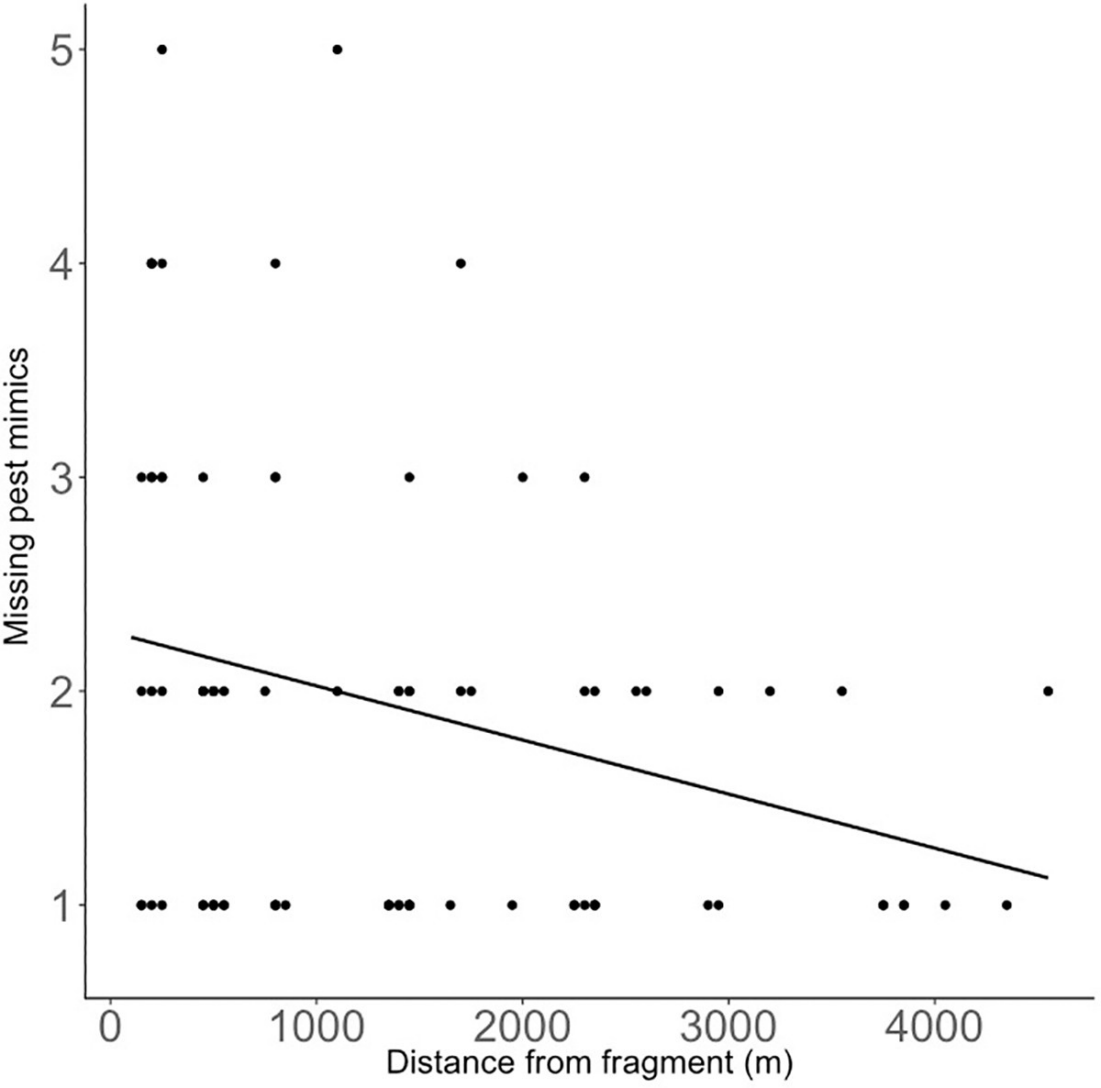
The relationship between missing pest mimics and distance of crops from forest fragments. Predicted lines are plotted from the model in S4 Table.

This pattern was broadly the same for missing pest mimics. The number of missing pest mimics (as an index of predation rate on pest mimics) increased with increased insect-eating birds but again, this positive relationship becomes weaker further from the forest fragments (Table 2, Fig 5). We found a strong average positive main effect of insect-eating bird abundance when not controlling for distance (S3 Table) and a strong negative effect of distance when not controlling for insect-eating bird abundance (S4 Table, Fig 6).

**Fig 7:**
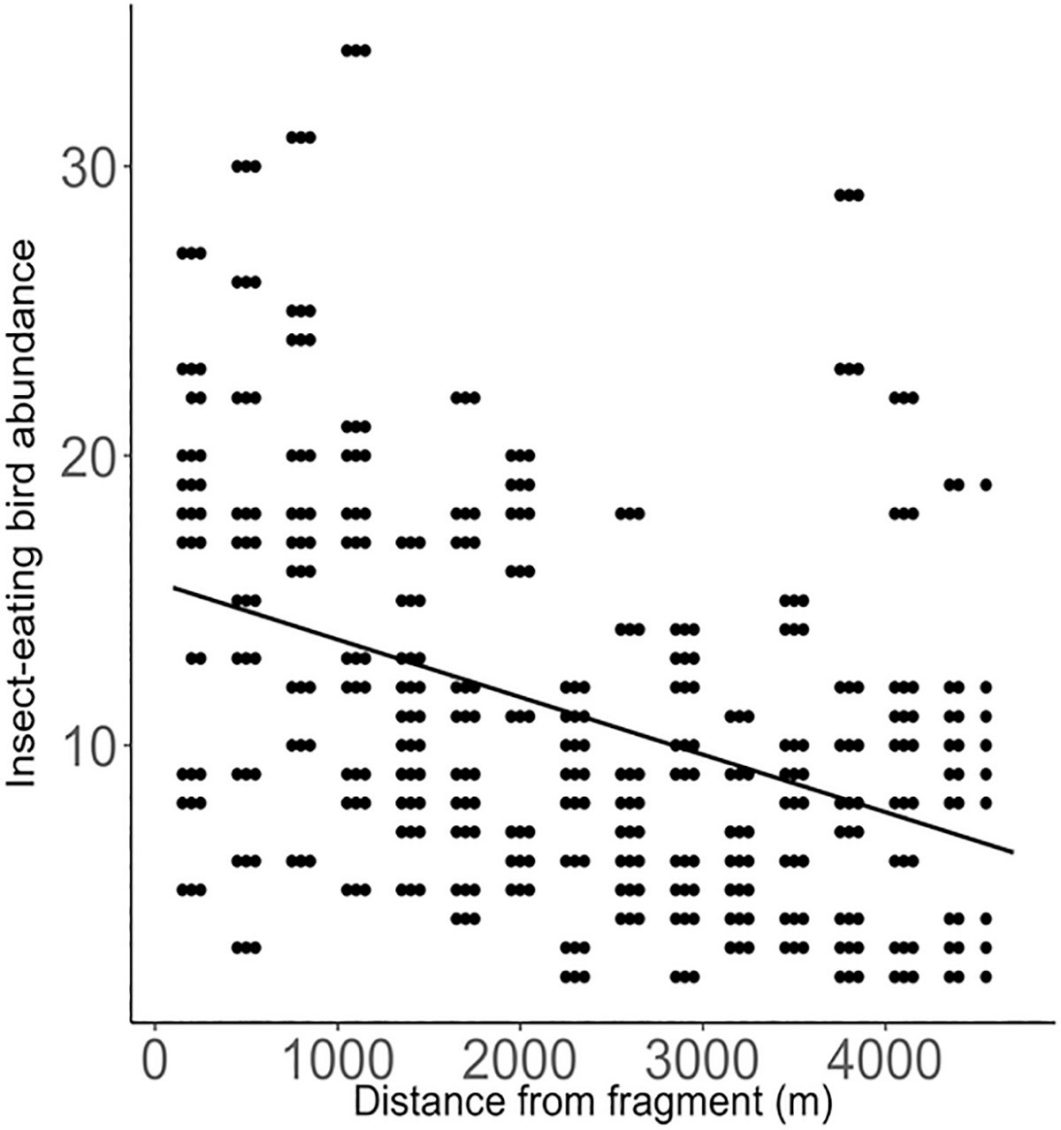
The relationship between insect-eating bird abundance and distance from forest fragments. Predicted lines are plotted from the model in Table 3.

The abundance of insect-eating birds decreased as distance from a forest fragment increased (Table 3, Fig 7).

**Table 3:** Model 4. The relationship between insect-eating bird abundance and forest proximity.

Variables	Estimate	SE	*t*	*p*
(Intercept)	15.48	1.23	12.55	**<0.001**
Distance	−0.0019	0.00047	−4.0068	**<0.001**

*Note*. Model: Insect-eating bird abundance = distance, random = ~1|section, method = "ML". Significant p–values are given in bold.

## Discussion

Our study fills crucial knowledge gaps around the role of birds in pest control and insight into the value to farmers of their maintaining trees in subsistence farm landscapes by providing the first quantitative evidence for insectivorous birds probably providing pest control services to crops in West African subsistence farmlands. We found that bird predation on pest mimics significantly increased with increasing abundance of insect-eating birds. Our results suggest that birds within this Nigerian forest-agricultural landscape make a strong contribution towards ecosystem services [34]. This is particularly important for subsistence farmers, who have limited economic access to external resources [16] and are constrained by the cost and availability of insecticides [70, 71].

Our observations are in line with other studies in tropical agroforestry systems [9, 41, 58, 72], which showed a strong correlation between insectivorous bird abundance and bird predation on artificial mimics. Together, these results counter common perceptions by farmers of birds being only pests of crops [10, 73].

We carried out our experiments during the rainy season, which coincides with the breeding season of many of the bird species we recorded visiting farms, and our findings suggest that birds were responsible for most of the predation on the pest mimics. Often birds show increased dependence on insects during their breeding season, with caterpillars comprising between 20–90% of diets at this time [74–76]. This is the case in several of the common foragers at our site, e.g., the African stonechat, bronze mannikin, common fiscal, little bee-eater and the family Cisticolidae [72, 77, 78]. Moreover, the common bulbul, an extremely common species recorded in this study switches from a predominantly frugivorous to insectivorous diet during the breeding season [78, 79]. Whinchats, European migrants [80], were also common at Ngel Nyaki during this study. Whinchats are insectivorous [81] and use farmland habitats extensively [82]. These suggests that birds may be more effective in pest control during the rainy season than the dry season.

We found proximity to the forest was an important variable in explaining both bird abundance and the intensity of bird predation on pest mimics. Our result suggests that insect-eating birds using the forest fragments also forage in farmlands and eat insect pests. Many of the bird species in our study are forest edge species [72, 83, 84], using forest trees for foraging, breeding and roosting [85, 86]. In this way forest fragments, by sheltering insect-eating birds as well as other mobile insect predators, may contribute to pest control services [8, 87], although the specific influence of a forest fragment is likely to depend on a range of variables such as its size, quality, degree of isolation and time since fragmentation [88].

Our findings indicates that crops positioned close to forest may have an advantage over crops grown away from the forest, as has been demonstrated for coffee plantations in the Neotropics [8, 36] and elsewhere [89]. Moreover, they suggest that forest patches on the Mambilla plateau should be conserved, rather than destroyed for planting crops or for grazing cattle, as is happening now. This will provide suitable semi-natural habitats for a wide variety of bird species, while providing valuable ecosystem services to the farmlands. In addition, degraded forest areas may be restored through tree planting to create additional semi-natural habitats for birds. Doing this will increase landscape complexity which should positively influence bird abundance [11], increase bird-mediated pest control [90], and allow the spill-over of other ecosystem services from the natural habitats to farmlands [91]. There are additional benefits to promoting semi natural habitats: on the Mambilla plateau tree planting can directly benefit farmers by reducing soil erosion, preventing springs from drying out and rivers from flooding.

We demonstrated that birds may control pests of groundnuts and bambara nuts and this is of economic significance given that insect pests regularly reduce groundnut yields by 10–20% [92] and, in Nigeria, by up to 40%. Nigeria is the major producer of groundnuts in Africa [49, 92] and, there is currently a resurgence in interest in farming cash crops [93, 94]. It will be vital to transfer our findings to farmers and policy makers. For example, insectivorous birds could be lured onto farms by adding appropriate nesting boxes [73, 95–97]. Also, tree species attractive to birds can be identified and planted on farms. These strategies may enhance bird services to farmlands, particularly in areas where there are no forests.

Overall, our study is especially timely for Nigeria where human population is increasing at an unprecedented rate [2] and at the same time oil revenue is decreasing [98], so that subsistence farming is on the increase [16, 99]. This places forests at risk across Nigeria but also Africa [6, 100]. Moreover, the meagre income of subsistence farmers [16] limits their access to insecticides [70, 71], and at the same time as argued above [29] integrated pest management is far preferable to chemical control [11, 90]. Both understanding and demonstrating the value of birds as biocontrol agents of crop pests, and the value of forest patches to these birds, is a first step in both improving a livelihoods and conserving forest habitat.

Our study did not look at the effect of forest size on insectivorous bird populations but given our findings that forests are important in providing the ecosystem service of pest control, more applied research in this area would be helpful in order to advise farmers on farm design. Moreover, it may be that planting stands of trees would be beneficial, as has been shown in other agricultural landscapes [3, 101]. Also, other factors that we did not consider in this study can affect pest predation. For example, identification of insect pest species in the farmlands [102], and time of year [72], all influence the ecosystem service of pest control by birds.

Our study in demonstrating that birds are important in providing insect pest control has set the scene for more research. Future research should scale up our investigations so that we quantify the value of forest fragments in terms of pest control; identifying which bird species are most important to be integrated into pest management programs, identifying the proportion of pests removed by birds, and the value of different sized forest fragments in terms of the number of insectivorous birds they harbour, e.g., see Karp et al. [8].

## Supporting information

S1 Table Model 2A: The relationship between bird attack marks on pest mimics and insect-eating birds. (PDF)

S2 Table Model 3A: The relationship between bird attack marks and forest proximity. (PDF)

S3 Table Model 2B: The relationship between number of missing pest mimics and insect-eating bird abundance. (PDF)

S4 Table Model 3B: The relationship between missing pest mimics and forest proximity. (PDF)

S5 Table Bird species recorded at experimental plots. (PDF)

S1 Data Manuscript data. (XLSX)
