# Supplementary material for: Pest-removal services provided by birds on subsistence farms in south-eastern Nigeria
Source: PLoS One. 2021 Aug 9;16(8):e0255638. doi: 10.1371/journal.pone.0255638 (PMC8351970; doi:10.1371/journal.pone.0255638)
Supplement: S1 Table — (PDF) [file pone.0255638.s001.pdf]

S1 Table. Model 2A: The relationship between bird attack marks on pest mimics and insect-eating birds.

Variables	Estimate	SE	*t*	*p*
(Intercept)	1.73	0.20	8.52	**<0.001**
Insect-eating bird abundance	0.13	0.015	8.45	**<0.001**

Note. Model; Bird attack marks = insect-eating birds, random = ~1|section, method = "ML". Significant p–values are given in bold
