# Supplementary material for: Pest-removal services provided by birds on subsistence farms in south-eastern Nigeria
Source: PLoS One. 2021 Aug 9;16(8):e0255638. doi: 10.1371/journal.pone.0255638 (PMC8351970; doi:10.1371/journal.pone.0255638)
Supplement: S2 Table — (PDF) [file pone.0255638.s002.pdf]

S2 Table. Model 3A: The relationship between bird attack marks and forest proximity.

Variables	Estimate	SE	*t*	*p*
(Intercept)	5.04	0.25	20.43	**<0.001**
Distance	−0.00088	0.000095	−9.29	**<0.001**

Note. Model; Bird attack marks = distance, random = ~1|section, method = "ML". Significant p–values are given in bold.
