# Supplementary material for: Pest-removal services provided by birds on subsistence farms in south-eastern Nigeria
Source: PLoS One. 2021 Aug 9;16(8):e0255638. doi: 10.1371/journal.pone.0255638 (PMC8351970; doi:10.1371/journal.pone.0255638)
Supplement: S3 Table — (PDF) [file pone.0255638.s003.pdf]

S3 Table. Model 2B: The relationship between number of missing pest mimics and insect-eating bird abundance.

Variables	Estimate	SE	*t*	*p*
(Intercept)	1.20	0.27	4.45	**0.001**
Insect-eating bird abundance	0.045	0.017	2.61	**0.011**

Note. Model; Missing pest mimics = insect-eating birds, random=~1|section, method="ML". Significant p–values are given in bold.
