# Supplementary material for: Pest-removal services provided by birds on subsistence farms in south-eastern Nigeria
Source: PLoS One. 2021 Aug 9;16(8):e0255638. doi: 10.1371/journal.pone.0255638 (PMC8351970; doi:10.1371/journal.pone.0255638)
Supplement: S4 Table — (PDF) [file pone.0255638.s004.pdf]

S4 Table. Model 3B: The relationship between missing pest mimics and forest proximity.

Variables	Estimate	SE	*t*	*p*
(Intercept)	2.34	0.32	7.32	**0.000**
Distance	−0.00026	0.00012	−2.23	**0.029**

Note. Model: Missing pest mimics = distance, random = ~1|section, method = "ML". Significant p–values are given in bold.
