# Supplementary material for: Pest-removal services provided by birds on subsistence farms in south-eastern Nigeria
Source: PLoS One. 2021 Aug 9;16(8):e0255638. doi: 10.1371/journal.pone.0255638 (PMC8351970; doi:10.1371/journal.pone.0255638)
Supplement: S5 Table — (PDF) [file pone.0255638.s005.pdf]

S5 Table: Bird species recorded at experimental plots

Common names	Family name	Scientific name	TFG
African Blue-flycatcher	Monarchidae	*Elminia longicauda*	insectivorous
African Dusky Flycatcher	Muscicapidae	*Muscicapa adusta*	insectivorous
African Moustached Grass-warbler	Macrosphenidae	*Melocichla mentalis*	insectivorous
African Paradise-flycatcher	Monarchidae	*Terpsiphone viridis*	insectivorous
African Pipit	Motacillidae	*Anthus cinnamomeus*	insectivorous
African Plain Martin	Hirundinidae	*Riparia paludicola*	insectivorous
African Reed-warbler	Acrocephalidae	*Acrocephalus (scirpaceus) baeticatus*	insectivorous
African Shrike-flycatcher	Vangidae	*Megabyas flammulatus*	insectivorous
African Thrush	Turdidae	*Turdus pelios*	insectivorous
African Yellow White-eye	Zosteropidae	*Zosterops senegalensis*	insectivorous
Bamenda apalis	Cisticolidae	*Apalis bamendae*	insectivorous
Banded Martin	Hirundinidae	*Neophedina cincta*	insectivorous
Bangwa Warbler	Sylviidae (Locustellidae)	*Bradypterus (lopezi) bangwaensis*	insectivorous
Black Bee-eater	Meropidae	*Merops gularis*	insectivorous
Black Crake	Rallidae	*Zapornia flavirostra*	omnivorous
Black Saw-wing	Hirundinidae	*Psalidoprocne pristoptera*	insectivorous
Black-capped Woodland Warbler	Sylviidae	*Phylloscopus herberti*	insectivorous
Black-collared Apalis	Cisticolidae	*Oreolais pulcher*	insectivorous
Black-crowned Tchagra	Malaconotidae	*Tchagra senegalus*	insectivorous
Blue-breated Bee-eater	Meropidae	*Merops variegatus*	insectivorous
Blue-shouldered Robin-chat	Muscicapidae	*Cossypha cyanocampter*	insectivorous
Broad-tailed Grassbird	Sylviidae (Locustellidae)	*Schoenicola platyurus*	insectivorous
Bronze mannikin	Estrildidae	*Spermestes cucullata*	granivores
Brown-chested Lapwing	Charadriidae	*Vanellus superciliosus*	insectivorous
Brown-crowned Tchagra	Malaconotidae	*Tchagra australis*	insectivorous
Brown-rumped Bunting	Emberizidae	*Emberiza affinis*	insectivorous
Brown-throated Wattle-eye	Platysteiridae	*Platysteira cyanea*	insectivorous
Buff-throated Apalis	Cisticolidae	*Apalis rufogularis*	insectivorous
Cattle Egret	Ardeidae	*Bubulcus ibis*	insectivorous
Chubb's Cisticola	Cisticolidae	*Cisticola chubbi*	insectivorous

Common (African) Stonechat	Muscicapidae	*Saxicola torquatus*	insectivorous
Common Bulbul	Pycnonotidae	*Pyconotus barbatus*	generalist
Common Fiscal	Laniidae	*Lanius collaris*	insectivorous
Common Whitethroat	Sylviidae	*Sylvia communis*	omnivorous
Croaking cisticola	Cisticolidae	*Cisticola natalensis*	insectivorous
Diederik Cuckoo	Cuculidae	*Chrysococcyx caprius*	insectivorous
Doubled spurred Francolin	Phasianidae	*Ptemistis bicalcaratus*	generalist
Dusky Crested-flycatcher	Stenostiridae	*Elminia nigromitrata*	insectivorous
Eurasian Wryneck	Picidae	*Jynx torquilla*	insectivorous
European Pied Flycatcher	Muscicapidae	*Ficedula hypoleuca*	insectivorous
Familiar Chat	Muscicapidae	*Oeananthe familiaris*	insectivorous
Fine-spotted Woodpecker	Picidae	*Campethera punctuligera*	insectivorous
Flappet Lark	Alaudidae	*Mirafra rufocinnamomea*	insectivorous
Green Hylia	Scotocercidae	*Hylia prasina*	insectivorous
Green Longtail	Cisticolidae	*Urolais epichlorus*	insectivorous
Grey Apalis	Cisticolidae	*Apalis cinerea*	insectivorous
Grey Tit-flycatcher	Muscicapidae	*Myioparus plumbeus*	insectivorous
Grey Woodpecker	Picidae	*Dendropicos goertae*	insectivorous
Grey-backed (Bleating) Camaroptera	Cisticolidae	*Camaroptera brachyura*	insectivorous
Horus Swift	Apodidae	*Apus horus*	insectivorous
Large Rock Martin	Hirundinidae	*Ptyonoprogne fuligula*	insectivorous
Leaf-love	Pycnonotidae	*Pyrrhurus scandens*	insectivorous
Lesser Honeyguide	Indicatoridae	*Indicator minor*	insectivorous
Levaillant's Cuckoo	Cuculidae	*Clamator levaillantii*	insectivorous
Little Bee-eater	Meropidae	*Merops pusillus*	insectivorous
Little Egret	Ardeidae	*Egretta garzetta*	insectivorous
Little Greenbul	Pycnonotidae	*Eurillas virens*	generalist
Long-billed Pipit	Motacillidae	*Anthus similis*	insectivorous
Lyre-tailed Honeyguide	Indicatoridae	*Melichneutes robustus*	insectivorous
Marsh Tchagra	Malaconotidae	*Bocagia minuta*	insectivorous
Northern Black-flycatcher	Muscicapidae	*Melaenomis edolioides*	insectivorous
Northern Crombec	Macrosphenidae	*Sylvietta brachyura*	insectivorous
Northern Puffback	Malaconotidae	*Dryoscopus gambensis*	insectivorous

Northern Wheatear	Muscicapidae	*Oenanthe oenanthe*	insectivorous
Oriole Warbler	Cisticolidae	*Hypergerus atriceps*	insectivorous
Pale-eyed Black Tit	Paridae	*Melaniparus guineensis*	insectivorous
Pectoral-patch Cisticola	Cisticolidae	*Cisticola brunnescens*	insectivorous
Pied Crow	Corvidae	*Corvus albus*	omnivorous
Plain-backed Pipit	Motacillidae	*Anthus leucophrys*	insectivorous
Red-faced Cisticola	Cisticolidae	*Cisticola erythrops*	insectivorous
Red-shouldered Cuckooshrike	Campephagidae	*Campephaga phoenicea*	insectivorous
Red-tailed Bristlebill	Pycnonotidae	*Bleda syndactylus*	insectivorous
Rufous-naped Lark	Alaudidae	*Mirafra africana*	insectivorous
Rufous-necked Wryneck	Picidae	*Jynx ruficollis*	insectivorous
Scaly Francolin	Phasianidae	*Ptemistis squamatus*	generalist
Sedge Warbler	Acrocephalidae	*Acrocephalus schoenobaenus*	insectivorous
Senegal Coucal	Cuculidae	*Centropus senegalensis*	insectivorous
Senegal Eremomela	Cisticolidae	*Eremomela pusilla*	insectivorous
Short-winged Cisticola	Cisticolidae	*Cisticola brachypterus*	insectivorous
Snowy-Crowned Robin-chat	Muscicapidae	*Cossypha niveicapilla*	insectivorous
Sooty Chat	Muscicapidae	*Myrmecocichla nigra*	insectivorous
Square-tailed Drongo	Dicruridae	*Dicrurus ludwigii*	insectivorous
Sun lark	Alaudidae	*Galerida modesta*	insectivorous
Tawny-flanked Prinia	Cisticolidae	*Prinia subflava*	insectivorous
Tree Pipit	Motacillidae	*Anthus trivialis*	insectivorous
Velvet-mantled Drongo	Dicruridae	*Dicrurus modestus*	insectivorous
Wattled Lapwing	Charadriidae	*Venellus senegallus*	insectivorous
Western Yellow Wagtail	Motacillidae	*Motacilla flava*	insectivorous
Whinchat	Turdidae	*Saxicola rubetra*	insectivorous
Whistling Cisticola	Cisticolidae	*Cisticola lateralis*	insectivorous
White-chinned Prinia	Cisticolidae	*Schistolais leucopogon*	insectivorous
White-crowned Robin-chat	Muscicapidae	*Cossypha albicapillus*	insectivorous
White-spotted Flufftail	Rallidae	*Sarothrura pulchra*	insectivorous
Willow Warbler	Phylloscopidae	*Phylloscopus trochilus*	insectivorous
Yellow-footed Honneyguide	Indicatoridae	*Melignomon eisentrauti*	insectivorous
Yellow-throated Longclaw	Motacillidae	*Macronyx croceus*	insectivorous
Zitting Cisticola	Cisticolidae	*Cisticola juncidis*	insectivorous
